# A Preference Based Measure of Complementary Feeding Quality: Application to the Avon Longitudinal Study of Parents and Children

**DOI:** 10.1371/journal.pone.0076111

**Published:** 2013-10-14

**Authors:** Murthy N. Mittinty, Rebecca K. Golley, Lisa G. Smithers, Laima Brazionis, John W. Lynch

**Affiliations:** 1 Discipline of Public Health, The University of Adelaide, Adelaide, Australia; 2 Sansom Institute for Health Research, University of South Australia, Adelaide, Australia; 3 School of Social and Community Medicine, University of Bristol, Bristol, England; Queensland University of Technology, Australia

## Abstract

This paper presents the development of the Complementary Feeding Utility Index (CFUI), a composite index aimed to measure adherence to infant feeding guidelines. Through an axiomatic characterization this paper shows the advantages in using the CFUI are the following: it avoids the use of arbitrary cut-offs, and by converting observed diet preferences into utilities, summing the score is meaningful. In addition, as the CFUI is designed to be scored continuously, it allows the transition from intake of beneficial foods (in low quantities) and intake of detrimental foods (in high quantities) to be more subtle. The paper first describes the rationale being the development of the CFUI and then elaborates on the methodology used to develop the CFUI, including the process of selecting the components. The methodology is applied to data collected from the Avon Longitudinal Study of Parents and Children to show the advantages of the CFUI over traditional diet index approaches. Unlike traditional approaches, the distribution of the CFUI does not peak towards mean value but distributes evenly towards the tails of the distribution.

## Introduction

In nutritional epidemiology summarizing multiple foods into a single diet score is useful for studying the association between diet and health. Several data-reduction methods have been used to characterize whole diets including where food intake is assessed against a dietary index [1]. Dietary indices are designed prior to analysis and usually reflect diet in terms of adherence to dietary guidelines (e.g. Healthy Eating Index), dietary variety (Dietary Variety Score), or dietary style (e.g. Mediterranean Diet Score) [1]–[3]. Several reviews have concluded that higher diet index scores are generally associated with better nutrient intakes and health outcomes [1], [3]–[5]. However, a number of issues have been raised regarding dietary index construction—in relation to their meaningfulness and use in predicting health outcomes [3]–[7]. For example, Waijers & Feskens [3]question whether it is appropriate to sum index components that have different units of measurement (e.g. where precent fat intake is added to servings per day of cereals) and measurement scale (e.g. breastfeeding “0–3 times/day” may be ranked as 1, but for eating meat “never/rarely” is ranked as 1). Also, while the appeal to base index components on food-based dietary guidelines is acknowledged [2], [3], Wijers & Feskens [3] raise concerns about loss of discriminating power when index components are scored against single cut-offs. Furthermore, the traditional way of scoring indices does not include information about the “distance” any individual behaviour may be compared with perfect adherence to guidelines. It does not reward individuals who have consistency across index components nor provide useful information for individuals with midrange scores [2].

The primary aim of the present work is to propose methodology for constructing a dietary index, which avoids some of the shortcomings of the measures currently in use. It is in part motivated by several reviews and critiques of current practice [2], [3], [8]. The CFUI characterizes complementary feeding quality and meets the following recommendations;

- Reflects current dietary guidelines [2], [3], [8]
- Is based on food choices and intake [3], [8]
- Scoring ranges are used rather than arbitrary cut-offs [3], [9]
- Allows for meaningful summing of scores, from components with different scales [9]
- Those with greater adherence across index components score higher than those who vary in adherence between components

Here we describe our two-step approach to developing the CFUI. Firstly, we draw on utility theory to identify relevant functions for converting an individual's food intake (i.e. food preferences) into utilities that represent the component scores of the index. Secondly, we use “*displaced ideal*” theory i.e. the Euclidian distance from ideal behaviour to reflect compliance with guidelines. The displaced ideal expresses utilities of components marginally [10]–[13]. This process allows one to map a multi-dimensional space into a single dimensional measure-free space, so that adding the scores from multiple variables is meaningful. An axiomatic approach is used to derive the measure. The development of the CFUI comprises the following steps:

- Determination of index components
- Computing the single utility values and
- Computing the total utility (i.e. computing the total index score)

The remainder of this paper is divided into four sections. In Section 2 we determine the index components and provide the theory for transforming the food preferences into utilities. In Section 3, we review the standard functions used for combining index components into a single-dimension and describe the modifications that allow us to develop an index which achieves the desirable properties listed above. In Section 4 we show the gains of the CFUI over current methods using data from the Avon Longitudinal Study for Parents and Children (ALSPAC).

## Methods

### Determination of index components

The first step in utility value analysis involves identification of the components to be included in the index. These are chosen using existing dietary guidelines and/or expert knowledge. We reviewed infant dietary guidelines [14], [15] and identified 14 components for the index show in Appendix S1 in File S1 [16].

### Method for computing utilities for individual components (Partial utilities)

In the second step, an individual's nutritional intake or preference is assigned a score for each of the 14 index components. In the traditional practice of index development, eating preferences of individuals with different units of measurement are converted to an arbitrary index component score (i.e “points”). For example, an optimal breastfeeding preference of ‘always’ is allocated 10 points, as is an optimal vegetable intake of 3 serves per day. The meaningfulness of summing ordinal component scores that are measured in different units has been questioned by various researchers [4], [9], [17]. Furthermore, in traditional index development, the intervals between index components are usually considered to be equal, which may not be a valid assumption. For the CFUI we moved away from the traditional practice in the sense that we converted the eating preference of individuals into utilities in a way where summing was appropriate and meaningful, as they are measure free.

We used the von-Neumann and Morgenstern utility theory [18] to develop a scale and measure free index. We selected a utility function *f*(*x*) to describe a respondent's preference between all states of a component and assigned a single number (a probability) to express the desirability of a state. For example, it is possible to compare breast feeding with probability 1, to vegetable exposure with probability *p*, or no vegetable exposure with probability 1-*p*. By adjusting *p*, the point at which vegetable exposure becomes preferable defines the ratio of the utilities of the two index components.

In practice, an individual's dietary preference is converted into a utility for each component on the index, *u_ic_*. Partial utilities are measured on a cardinal scale with the range being 0 (non-adherence to guidelines) to 1 (complete adherence to guidelines). By converting actual food intake to probabilities we created a measure-free measure that can be summed and compared, rather than assigning a score based on arbitrary cut-off values.

Partial utilities are calculated from the food consumption set, *X*, where each component 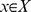 is a vector comprising the preferences of each component on the real line, 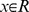, in our example the *X* is a 14 dimensional space i.e 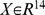. The goal is to find a function *f*(*x*) that best represents the observed preference pattern.

In order to construct partial utilities for each component, we make the following assumptions on people's preferences. We denote the preferences relation by “
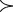
”.

1. Completeness: for any two consumption states *s*_1_ and *s*_2_ 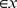, either *s*_1_ 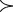 *s*_2_, or *s*_2_ 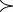 *s*_1_ exists and, therefore all states can be compared with one another.
2. Transitivity: for any three consumption states *s*_1_, *s*_2_, *s*_3_ 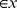, if s_1_ is preferred to *s*_2_ and *s*_2_ is preferred to *s*_3_ then *s*_1_ is preferred to *s*_3_.
3. Continuity: assumes that there are utilities in between complete adherence and non-adherence to dietary guidelines.
4. Monotonicity: this means that a consumption state which assigns a higher probability to a preferred outcome will score higher than one which assigns a lower probability to a preferred outcome, as long as the other outcomes remain unchanged. This case refers to a strict preference of an outcome.
5. Substitution: preferences are linear with respect to probability.

### Appropriate choice of function for transforming preferences to utilities

Conversion of Breastfeeding (BF) preference into partial utilities was straight forward and was derived from data. However, other index components were more complex and therefore required the use of an exponential distribution and three parameter Pareto distribution as explained below.

For the component BF duration, the function that we used to assign a real number which lies between 0 and 1 for every respondent in a way that captures the respondents' preference is; 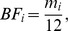
where *m_i_* is the number of months of breastfeeding by the *i* ^th^ respondent. Here the denominator 12 was chosen as this is the optimal BF duration recommended in current infant feeding guidelines [14], [15]. For example, individual breastfeeding for six months has a probability of 0.5.

Similarly Fed on Demand (FD) was a categorical variable, with *k* categories. The utility function for this is defined as 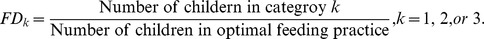
We choose the denominator to be the number of children in optimal feeding practice, in order to comply with the current dietary guidelines. Five other index components, including-exposure to iron rich cereals, introduction to cow's milk, exposure to tea, age of introduction to lumpy foods and meal frequency were also categorical variables and hence we used a similar utility function to FD to convert those preferences into utilities.

For the Protein Food Variety (PFV) component, preference options were consumption of one, two or three types of protein foods at the age of six months and for this we used an exponential utility function to reflect current guidelines. We would expect the utility function to show an increasing adherence to guidelines as the quality of food intake increases [14], [15], [19] and an exponential utility function reflects this criterion. Hence for PFV the function used was 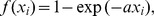
where ‘*a*’ is a positive constant that represents the degree of risk aversion and *x_i_* is the protein food variety for the *i* ^th^ individual, where risk aversion is defined as greater adherence to guidelines.

Similar to PFV we assumed the distribution for Timing of Solids Introduction, exposure to Vegetables and Fruit Consumption to be exponential.

For Sugary Drinks (SD) the utility function needed to show a decreasing score as the consumption of the number of SDs increased. In order to apply this principle we had to select a special function that satisfied four conditions:

- *f*(*x*) is concave downwards; i.e. 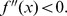 This property is referred to risk aversion in that it implies that for the intervals where the computed value of 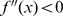 the function *f*(*x*) is concave downwards. Concave downwards utility also meant that (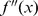) is a decreasing function for the consumption of sugary drinks, i.e. greater intake of SD reflect poorer adherence to guidelines.
- The absolute risk aversion decreases as consumption decreases, in other words, risk increases as consumption of SD increase. Absolute risk aversion is measured by 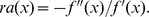 The *ra*(*x*) function can be seen as percentage change in single utility. Decrease in absolute risk means that the percentage change in single utility is itself decreasing.
- *f*(*x*) is bounded above and below, i.e. there are number *a* and *b* such that 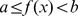 no matter how large *x* is. This criterion was necessary to keep very large values from dominating preferences. The lower bound was necessary to prevent very small values of preferences becoming negative.

Following Venter [20] a distribution that satisfies the above conditions is a three parameter Pareto distribution 
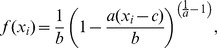
where *a*, *b*, and *c* are the shape, scale and size parameters [20]. Similarly we used a double bounded Pareto distribution to represent preference distribution for the index component Energy-Dense Nutrient–Poor foods. Parameters *a*, *b*, and *c* are derived from the data.

### Method for combining the utility

The final step was to define the method to combine components into a total index score. Currently in the nutrition literature, an overall index score is a simple sum of the partial utilities [3], [9]–[11], [13], [21]. However, these methods may not be appropriate for reflecting diet quality. For example, let us assume a two-component index comprised of breastfeeding and vegetable consumption with both components equally weighted. Now, let us assume that a score of 50 is given to both components for person *j* while for person *k*, their scores were zero and 100 respectively for the two components. Under the summing of scores approach both individuals get the same score suggesting that both are doing well overall. This is because linearity assumes that component scores are interchangeable. That is, an increment in one criterion at any value can be substituted by an equal decrement in another indicator at any other value [22]–[24].

However, we would hypothesize that the diet quality of person *j* (i.e. moderate adherence on both guidelines) may be better than the diet quality of person *k* (i.e. non-adherence and complete adherence to those guidelines respectively). To test this hypothesis we propose using a method based on “displaced ideal theory” developed by Zeleny [25]. The displaced ideal theory is based on the notion that consistency across scores is preferred. In this case the scoring method should align with our qualitative assessment that person *j* has a ‘better’ diet than person *k*.

### Axiomatic characterization of CFUI

This section presents six intuitive properties that a measure of diet quality should satisfy.

#### Normalization

A CFUI should have a minimum and a maximum and 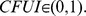 at its minimum *CFUI* = 0 indicating no adherence in all 14 components; and its maximum *CFUI* = 1, indicating a completer adherence to all 14 guidelines.

#### Anonymity

A CFUI should be indifferent to swapping of values across components. With two people *j* and *k*, this would mean that *CFUI_j_ = CFUI_k_* if the values are interchangeable across, for example, seven components and remained same on the other seven components.

#### Monotonicity

A CFUI should be greater if the index value in one component is greater with index values remaining constant in all other components. With two people *j* and *k*, this would mean that index values remain the same in two components and different in all others then 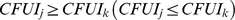 if and only if 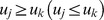.

#### Proximity

A CFUI should be such that a greater value indicates that it is closer to the ideal point, which is complete adherence to dietary guidelines. For two persons *j* and *k*, with Euclidian distance from the ideal indicated by *d_j_* and *d_k_* respectively then 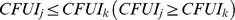 if and only if 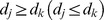.

#### Uniformity

A CFUI should be such that for a given mean index value, *μ*, a greater (or smaller) variation across dimensions, *σ*, should indicate a smaller (greater) total value. For two persons *j* and *k*, if 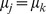 and 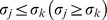 then 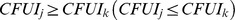. This is to assure that adherence to dietary guidelines is balanced or uniform across all components.

#### Signalling

A measure of CFUI should be such that as values shift from their initial position, the direction and magnitude of the change is signalled. In addition it should indicate a unique optimal path to reach the ideal value or higher value. That is, there exists one and only one distance 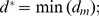 *m* =  possible paths.

### Displaced Ideal

The concept of ‘displaced ideal’ proposed by Zeleny [25] is based on the principle that a better configuration of partial utilities should have a higher overall score, i.e. be closer to the ideal.

Let *X* denote a set of all index components, i.e. 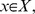 let 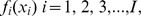 be the functions used to compute the utilities, now let 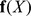 be the vector of all partial utilities 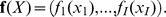
We can now state the multi attribute decision making problem simply as 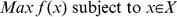
(1)which mathematically represents the vector function maximization problem.

One possible approach to solve expression (1) is through a direct assessment of the overall utility function, say, 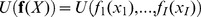
(2)As described in Zeleny [25] construction of *U* is complex. Although *U* is not known explicitly it can be safely assumed to be a real value, monotone function is each argument 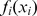, and possibly reflecting the conventional decreasing marginal rates of substitution property. Under such conditions it can be shown that at least one solution at which equation (2) achieves its maximum over *X* is non-dominated [25]. This non-dominated solution is called an effective solution or Pareto-optimal solution [26].

Let each individual component of 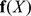 have maximum score of some 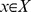, say 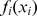 reaches its maximum at 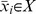, we can write: 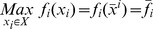
(3)Then 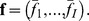 can be defined as the “ideal point”, a vector of all maximum feasible values attained by individual functions on X. So, if there would exist 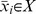, such that 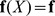 then the solution 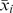, would be also the maximum reached by any increasing utility function *U*. There would be no decision problem. Such an ideal solution is however infeasible. On the other hand, instead of maximizing the solution, because of the ideal point, the decision maker can try to find a solution that is “*as close as possible*” to the ideal point. Salukavadze et al. [26] describe several methods for solving multi-criteria optimization.

The fuzzy state (“*as close as possible*”) is more feasible and realistic than maximization of *U* for our application. Now, if we denote the degree of closeness of an 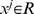, to 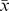, with respect to the *i*th component as 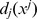 which has the properties: 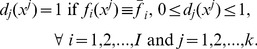
(4)The function 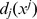 defines the metric space (*R^n^*, *d_i_*(*x^j^*)) called *L^p^* metric. Once again following Zeleny [25] and Salukvadze et al [26] it can be understood that a family of *L^p^* metric provides a range of geometric measures of closeness defined as: 
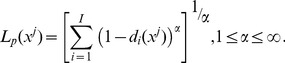
(5).

### Computing the CFUI using the displaced ideal method

For any measure based on distance, the first choice is the special case of Minkowaski distance, Euclidian distance. Following Nathan & Mishra [23], [24] in this paper we use the inverse Euclidian norm to compute the overall score. By normalizing to the scale of (0, 1), 0 being the least favoured (non-adherence) and 1 being most favoured (complete adherence), the ideal point would be defined by unity vector, *I* = (1, 1, …, 1). The method of combining the utilities using displaced ideal technique is given by, 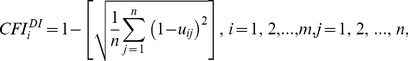
(6)assuming equal weights.

## Results

As an illustration, we applied out method to the complementary feeding period information collected in the Avon Longitudinal Study of Parent and Children (ALSPAC). ALSPAC recruited 14,541 pregnant women resident in Avon, UK with expected dates of delivery 1^st^ April 1991 to 31^st^ December 1992. This is the number of pregnancies for which the mother enrolled in the ALSPAC study and had either returned at least one questionnaire or attended a “Children in Focus” clinic by 19/07/99. Out of the initial 14,541 pregnancies, all but 69 had known birth outcome. Of these 14,472 pregnancies, 195 were twin, three were triplet and one was a quadruplet pregnancies meaning that there are 14,676 foetuses in the initial ALSPAC sample. The number of new pregnancies not in the initial sample that are currently represented on the built files is 542. Of the 542 additional pregnancies, 6 were twin, meaning that the number of additional children that need to be considered is 548. The total sample size for analysis using child-based questionnaire data collected after age seven is therefore 15,224. The questionnaire listed 43 food and beverage items at 6 months, increasing to 70 items at 15 months. Questionnaires also included information on breastfeeding, and formula feeding [27].

Often in nutritional epidemiology the common procedure of data reduction is to develop an index based on linear averaging. In this section we start with the traditional procedure and later demonstrate the advantages of using the new approach by the following the axiomatic characterization described in previous section. For an illustration, in the initial three sections we took a sample of random scores of two people from the data set. However, to illustrate the distributional gains we used the complete information available (9,276, missing cases excluded). Other detailed applications of the index, which are beyond the scope of this paper, are reported elsewhere [16].

### Linear averaging

For understanding the advancement of the new method we compare it with the traditional approach of index construction, linear averaging (LA).

In the traditional approach or LA, the underlying assumption is that the parameters are perfectly interchangeable [2]. That is under linear averaging, the increment in one component at any value can be substituted by an equal decrement in another indicator at any other value [22], [23]. This assumption is unquestionable when used in the case of the same parameters such as weights (kg) of children, or when items with similar scales are added to obtain a total value.

Use of perfect exchangeability of individual scores in the construction of a dietary index may not be appropriate. This is because, the individuals with high exposure to the components BF and V, and no exposure to SD are regarded as healthy compared to the ones whose exposure is the opposite. For the axiomatic comparisons we restrict our illustrations to two dimensional space using BF (breastfeeding) and V (vegetable consumption) variables. However, for the distributional comparison we use data from all 14 dimensions.

In the absence of reaching the ideal across components, the next best scenario would be to score uniformly across components (e.g. 0.8, 0.8, 0.8). Currently, under the LA approach people who score uniformly on all components are not rewarded any more than those who do not. However we demonstrate below that the DI method proposed in this paper rewards people who score uniformly, i.e., show greater variety in consumption.

**Figure 1:**
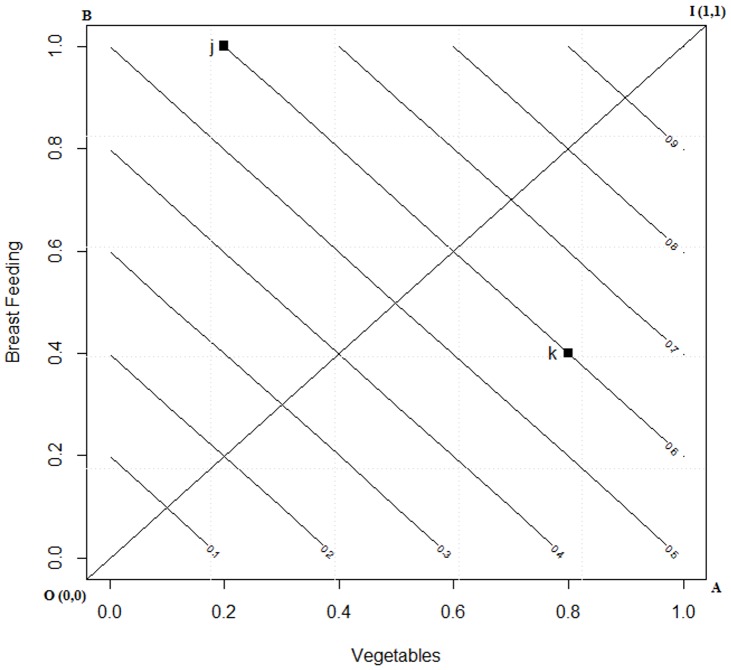
*ISO* curve for linear averaging in a two dimensional space. Footnote: J is an individual who score 0.8 and 0.4 on breast feeding and vegetable intake and K scores 0.2 and 1 then the CFI score computed under LA results in 0.6 on the ISO curve.

For a demonstration of the difference between a complementary feeding index computed using the LA and DI methods, let us consider two components BF and V, as it is easier to visualize in two dimensions. Using the Linear Averaging (LA) method, a Complementary Feeding Index (CFI) is given by: 
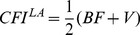
(7)The *iso*-*CFI^LA^* plot for the two dimensional space is given in Figure 1. Computations were performed using the “*R*” [28] statistical language.

From Figure 1 one may note that the *CFI* space *OAIB*, with origin *O* (0, 0), representing minimum adherence to guidelines for breast feeding and vegetable intake, and ideal adherence at *I* (1, 1) where both the indicators are at their maximum. Any random respondent will occupy a point in the space *OAIB*. The locus of all points having the same *CFI^LA^* score are shown as 45° inclined *iso-CFI^LA^* lines. It is apparent that *j* (0.2, 1) have the same *CFI^LA^* mean scores as that of *k* (0.8, 0.4) and hence are on the same plane. In other words they are considered to have the same diet quality.

### Displaced ideal

In a two dimensional *CFI* space, I denotes full adherence to a set of guidelines and a person completely adhering to the guidelines in all dimensions (BF = 1), and (V = 1). Following the theory above the *CFI^DI^* is given by 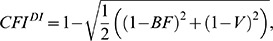
(8)where 
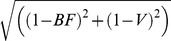
 is the Euclidian distance (*d_j_*) for the ideal, dividing by √2 normalizes it in two dimensional space and then subtracting the normalized distance from unity gives the inverse. Thus, for person *j* the shorter the distance from ideal, *d_j_*, the higher is the complementary feeding index score.

**Figure 2:**
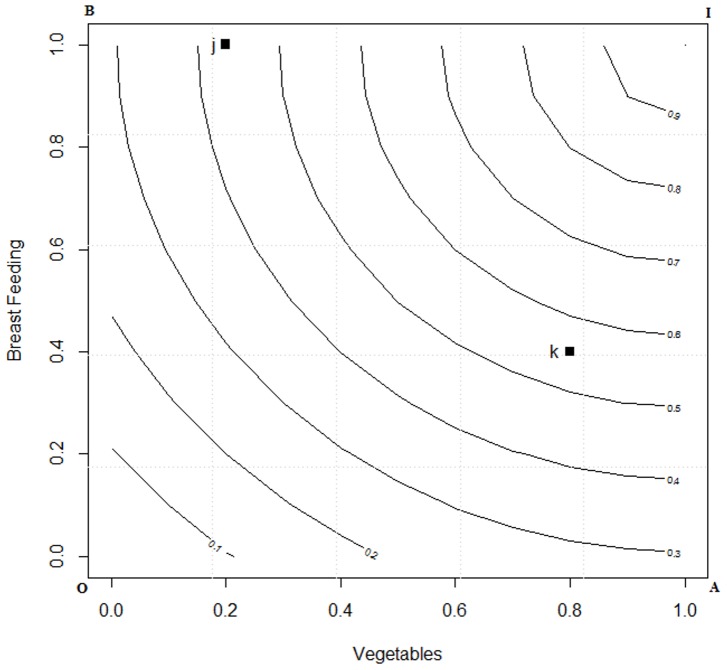
*ISO* curve for displaced ideal in a two dimensional space. Footnote: J is an individual who score 0.8 and 0.4 on breast feeding and vegetable intake and K scores 0.2 and 1 then the CFI score computed under DI results in 0.4 and 0.5 on the ISO curve.

The *iso-CFI^DI^* plot in two dimensional spaces is given in Figure 2. The CFI space presenting the two dimensions of BF and V and the two points *j* and *k* representing two persons diet preferences are kept the same as in Figure 1. Now in the *CFI^DI^* one might note that the place of *j* and *k* has changed. Earlier (Figure 1) was on the same plane, whereas now *k* has fared better than *j*. Thus with the application of DI theory we illustrate two points:

1. partial utilities (individual component scores) are not exchangeable, and
2. moderate adherence to each component of the index is preferred compared to high adherence to some and no adherence to others.

Table 1 shows the difference between *LA* and DI computation of the overall score which was graphically represented in Figures 1 and 2, these computations were carried out using equations (7) and (8). These equations are two special conditions of Minkowaski's distance, that is equation (7) is the first order distance measure and equation (8) is the second order distance measure. As one may observe the overall utility score for individual *j* under LA and DI is same. However, for individual *k* it may be observed that under DI theory, the overall score increases indicating that the distance from ideal is decreasing. Conversely, under the LA approach the increment remains invariant for the individual *k*. It may be observed from here that DI signals the individuals to progress along an ideal path which is based on the notion that an improvement in a component that has a lower value is more important than an equivalent improvement in a component that has a higher value.

**Table 1:** Comparison between overall scores obtained using LA and DI methods.

Respondent	*Breastfeeding*	*Vegetables*	*LA Score*	*DI Score*
*j*	0.2	1.0	0.6	0.6
*k*	0.8	0.4	0.6	0.684

### Axiomatic comparison between LA and DI

From the results it is clear that both the LA and DI methods satisfy axioms of Normalization, Anonymity and Monotonicity. However it is assessment of the axioms in terms of Proximity, Uniformity and Signalling that demonstrates advantages of DI over LA (Table 2).

**Table 2:** Comparison of the overall score computation using LA and DI under proposed axioms in two dimensions.

		Utilities			CFI Score			Direction of CFI score		
Axiom^1^	Person	*BF* 2	*V*	Distance	LA^3^	DI	Distance	*LA*	*DI*	Component
A	J	0.4	0.8	0.63	0.60	0.55	NI^4^	*j* = *k*	*j* = *k*	*BF_j_+V_k_ = BF_k_+V_j_*
	K	0.8	0.4	0.63	0.60	0.55	NI			*BF_j_ = V_k_; BF_k_ = V_j_*
										
M	J	0.8	0.7	0.36	0.75	0.75	NI	*j*>*k*	*j*>*k*	*BF_j_>BF_k_*
	K	0.4	0.7	0.65	0.55	0.53	NI			*V_j_ = V_k_*
	J	0.3	0.7	0.76	0.50	0.46	NI	*j*<*k*	*j<k*	*BF_j_<BF_k_*
	K	0.6	0.7	0.50	0.65	0.65	NI			*V_j_ = V_k_*
										
P	J	0.2	0.8	0.82	0.50	0.42	*d_j_*>*d_k_*	*j* = *k*	*j<k*	NI
	K	0.5	0.5	0.71	0.50	0.50	–			NI
	J	0.6	0.7	0.50	0.65	0.65	*d_j_*<*d_k_*	*j*<*k*	*j*>*k*	NI
	K	1.0	0.4	0.60	0.70	0.58	–			NI
Uniformity										
U-NU	J	0.5	0.5	0.70	0.50	0.50	*d_j_*<*d_k_*	*j* = *k*	*j*>k	*BF_j_+V_j_ = BF_k_+V_k_*
	K	0.7	0.3	0.76	0.50	0.46	–			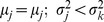
NU-U	J	0.8	0.6	0.45	0.70	0.68	*d_j_*>*d_k_*	*j* = *k*	*j<k*	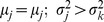
	K	0.7	0.7	0.42	0.70	0.70	–			
										
S	J	0.6	0.8	0.45	0.70	0.68	–	*j = k = m = n = o*	*j = m<k>n>o*	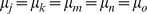
	K	0.7	0.7	0.42	0.70	0.70	*d_j_*>*d_k_*			
	M	0.8	0.6	0.45	0.70	0.68	*d_k_*<*d_m_*			
	N	0.9	0.5	0.51	0.70	0.64	*d_m_*<*d_n_*			
	O	1.0	0.4	0.60	0.70	0.58	*d_n_*<*d_o_*			

1 A—Anonymity, M—Monotonicity, P—Proximity U—Uniformity, NU—No-uniformity , S—Signalling.

2 BF—Breastfeeding , V—Vegetable.

3 LA—Linear Averaging, DI—Displaced Ideal.

4 NI— Computation Not of Interest.

#### Normalization

In both methods, the respondents are bounded by minimum, 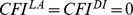 at the origin, and the maximum 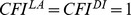, at the ideal 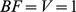. In the first two axioms Anonymity and Monotonicity we are not interested in the direction of *d_j_* and *d_k_* but are concerned with the direction of the contribution of the component (*BF*, *V*) score because our point of interest is to see which choice affects the total score. However, for the latter three components, Proximity, Uniformity, and Signalling we are interested in examining:

- how the distributions properties (mean and variance) affect the overall score computation and their placement from the ideal position
- how the overall score computation reflects the change in dispersion

From Table 2 the following conclusions can be made about LA and DI methods of combining the component scores.

#### Anonymity (A)

Both satisfy this. Interchanging the component scores does not alter the overall score of CFI. For two persons *j* and *k*, if the values across the components BF or V are interchanged, *BF_J_* = *V_k_* and *BF_k_* = *V_j_*, then 
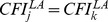
 and 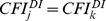.

#### Monotonicity (M)

This is also satisfied for the scores computed by both the methods. For two persons *j* and *k*, if the value is higher in one component and the other components remain the same *BF_j_*>*BF_k_* and *V_j_* = *V_k_*, then 
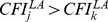
 and 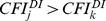. When the direction for the component utilities changes the overall score computed using LA and DI methods reflect such changes.

#### Proximity (P)

The DI method satisfies this but not the LA. For two respondents *j* and *k* with Euclidean distance from the ideal being such that *d_j_*>*d_k_* then 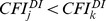, but it is possible to have 
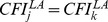
. In the second exercise, we noticed that, when *d_j_*<*d_k_* there is a possibility that the respondent with lower CFI score will be closer to the ideal point compared to the respondent with the higher CFI score, using the LA approach. Logically this does not make sense.

#### Uniformity (U)

the DI method satisfies this, but not the LA method. For two persons *j* and *k*, if 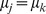 and 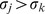 then 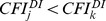, but 
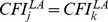
. The LA method is independent of the dispersion. But DI, on the contrary, will have minimum distance from the ideal if and only if the value lies on the line of equilibrium. The line of equilibrium is the locus of all local ideal positions, where a local ideal position is defined as the mean of the individual utilities. This line is drawn by joining all the means from origin *O* (0, 0) to the ideal position *I*(1,1).

#### Signalling (S)

**Figure 3:**
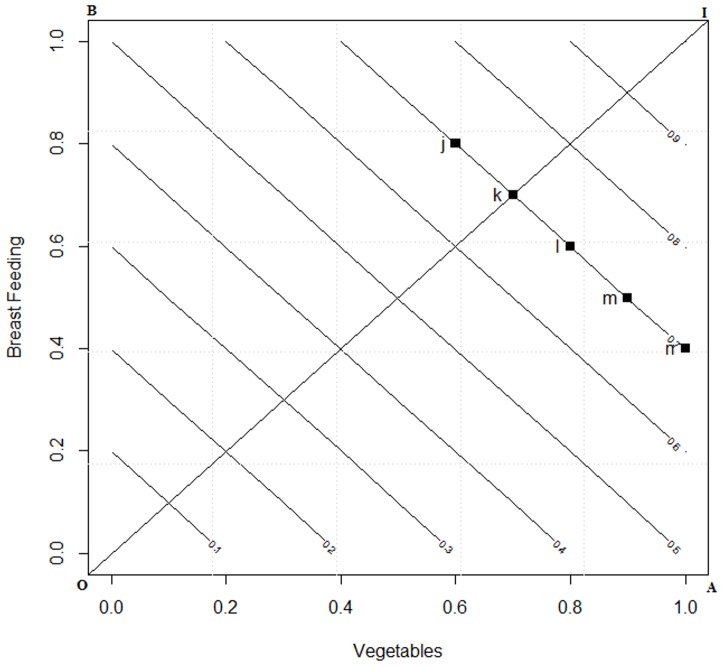
Signalling axiom applied to linear averaging. Footnote: Six individuals J, K, L, M, N, O whose distance from the line of equality.

**Figure 4:**
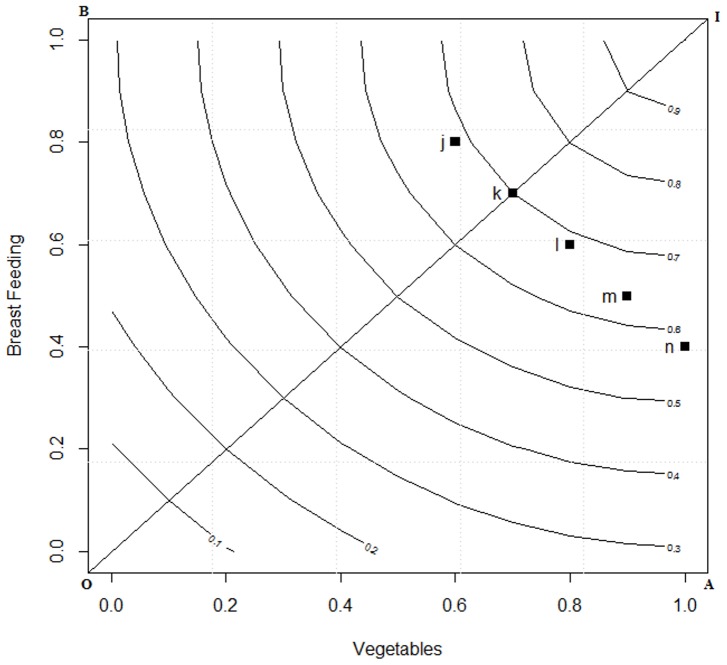
Signalling axiom applied to displace ideal. Footnote: Six individuals J, K, L, M, N, O whose distance from the line of equality.

the DI method satisfies this but not he LA method. For this exercise we took multiple scenarios where the diet utilities on the components BF and V are changed but the arithmetic mean of the utilities was kept constant. As can be seen from Figures 3 and 4 and Table 2 the CFI computed using the LA method did not change with a range of component scores. The *CFI^LA^* was stagnant on the *iso-CFI* curve and it is silent about a desirable path among the possibilities to improve diet quality. This graph clearly demonstrates the exchangeability effect, even when in reality it is not true. This stagnant behaviour is not helpful in making decisions about how to improve population diet quality.

However, in the DI method any change in the combination of the utilities the CFI scores also changed. The maximum of the CFI score was attained when the CFI scores falls on the 45° line (Figure 3 (0.7, 0.7)) which we call the line of equilibrium. This change in CFI by DI method reflects the idea that people who adhere to all dietary recommendations equally are more likely to be closer to the ideal diet than those who adhere extremely well on one and poorly on the other components. This finding leads to the assertion that between two paths, the path closer to the ideal path will have a higher *CFI^DI^*. Evidence of this assertion was derived independently and is the same as the one given in Mishra & Nathan [22]. The proof is reproduced with the permission from the author [24] in Appendix S2 in File S1.

Thus, displaced ideal satisfies all the axioms, whereas linear averaging satisfies only the first three, Normality, Anonymity, and Monotonicity. The failure arises because the linear averaging method assumes perfect substitutability across the two dimensions. Under perfect substitutability if 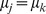 then 
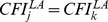
 even if 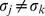 or 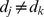. Further, it is least informative in indicating a desirable path among the infinite possibilities to improve on CFI. Since the scores computed using DI method do not assume perfect substitutability, any slight variability in the distribution shifts the distance from the ideal position, this now gives a unique ideal path to move from the actual position to a higher or lower position.

### Distributional comparison between the traditional and complementary feeding Index

**Figure 5:**
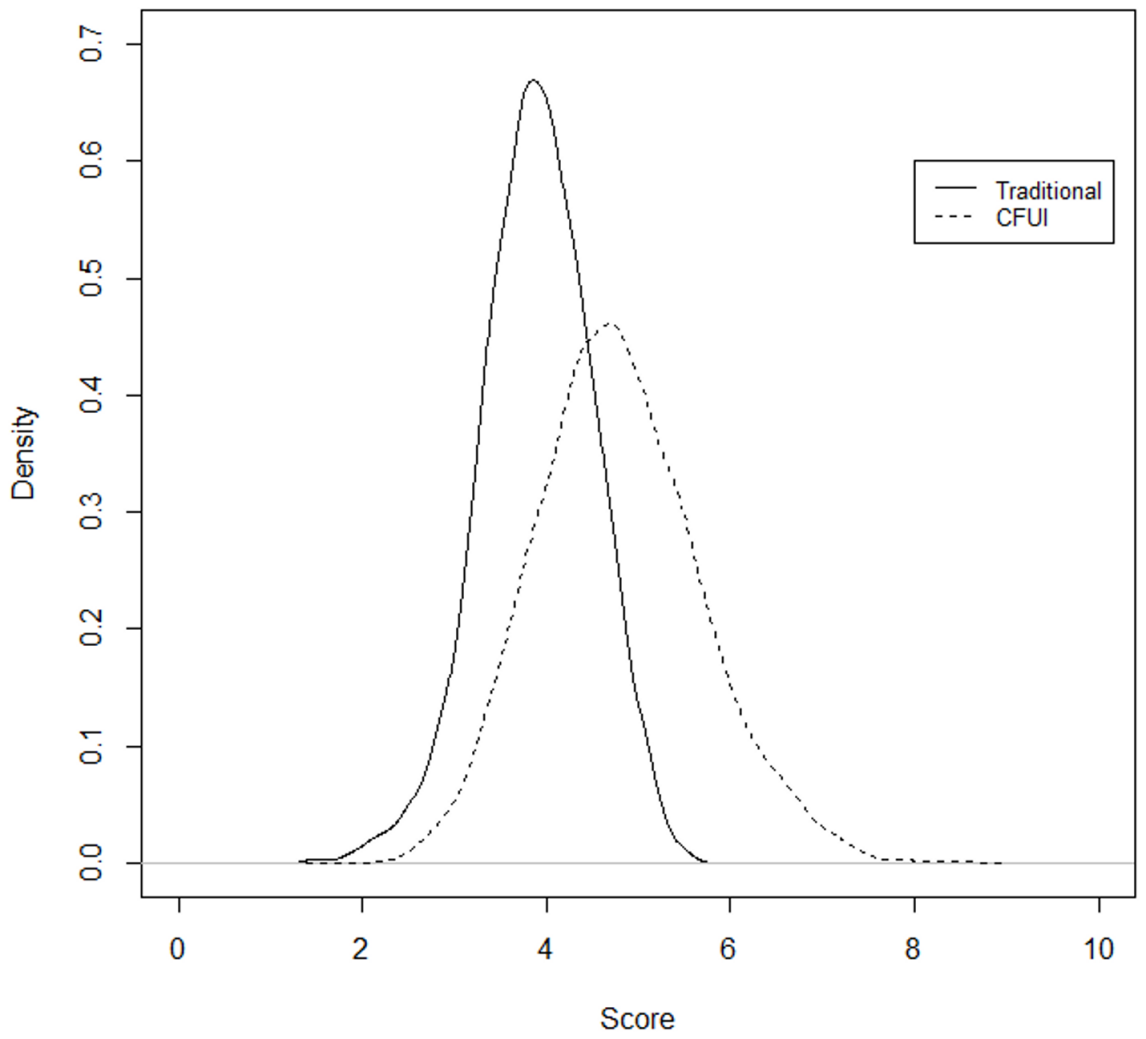
Comparison of traditional and displaced ideal based scoring of complementary feeding.

In seeking a distribution-sensitive index the objective is to not overlook at the actual distribution as done in linear averaging. Having said this, we do not claim that the distribution-sensitive measure captures the entire information contained in all the 14 separate individual components. Although we have improved the index to incorporate the variation into the score estimation, some loss of information remains. However, it will respond to the average value and in some ways to the dispersion around the average value. By this we mean a distribution sensitive measure will discriminate 1) how far the person's utility score is from the guideline value, and 2) how much change in the overall score is a result due to changed distribution pattern. Figure 5 provides a comparison between the distributions of the newly proposed method and the linear averaging. From the distributions one can observe that there is a complete location and scale shift with the complementary feeding index. The effect due to location shift and the shape shift was computed using the relative distribution methods proposed in Handcock & Morris [29] using the “*reldist*” package in R. The relative density provides a robust analysis of the differences between two distributions [29]. Moreover, it also allows examination and decomposition of the effect due to changes in location (median) and changes in shape. The measures developed by Handcock & Morris [29] are based on entropy, Kullback-Leibler divergence measure. Results from the analysis suggest that 94% of the effect is due to the location changes and only 6% of the effect is due to the change in shape. In context of the distribution of CFI these changes are especially important. For example comparing two distributions over time with the earlier distributions as the reference group, a simple location shift would indicate that everyone's CFI is larger (or smaller) by the same amount (or percentage). As there was evidence of divergence between distributions due to changes in shape, it is possible that polarization is occurring. To investigate this we used the median –relative polarization index following Handcock & Morris [29]. This measure is particularly useful because it is location adjusted, in this case for the median, which is an important link to the location and shape decompositions. Results show that the median relative polarization index value is positive indicating an increased polarization towards the tails. The high concentration of data around the mean value in the traditional method is due to the fact that 47.9% (4,452 ties in data out of 9,276 cases) of the data has ties values. However, in the CFI this problem is circumvented (66 ties out of 9,276) by the use of DI, thus appropriately representing the variation in the data.

## Conclusion

To date there has been no appropriate index for assessing diet quality during the complementary feeding period in developed countries. In this paper we have described the development of a preference-based index for measuring adherence to infant feeding guidelines. The index provides utility (preference) scores on a generic scale where non-adherence to guidelines  = 0 and perfect adherence  = 1. Such scoring systems have been used in the development of health indices [30]. Here we have attempted to make use of the utility theory in nutritional epidemiology. By converting the dietary intakes into utilities we have a technique where summing the utilities of index components that are measured on different scales is meaningful. The strength of the proposed methodology lies in the axioms used to derive it. This is an improvement from using arbitrary cut offs to derive component scores and assumptions of linearity to combine component scores. Moreover, we have proven geometrically that the method of combining scores using the displaced ideal has advantages over the simple linear averaging. By an axiomatic characterization and empirical verification we have shown that the DI method of combining the scores distinguishes between individuals who achieve midrange scores by scoring consistently across components compared with individuals whose midrange scores reflect adherence at the extremes across components. We feel this is an advantage because a very low score places the individual at greater risk of suboptimal nutrition on that component. Thus, the DI method of combining the scores captures uniformity and balanced behaviour across different nutritional dimensions, unlike the LA method where the exchangeability assumption is forced. The CFI also signals those components in which individuals are adhering and not adhering to guidelines. Currently used diet quality scores contain many subjective choices (e.g. cut-offs). By using a utility approach we provide a data driven method that is reproducible and not subjective in nature. In addition, by providing a scoring range for each component we provide a technique that allows judgement of intakes of foods or nutrients that are both beneficial and detrimental, thus making the transition from beneficial to detrimental more subtle. This is a major advance over the existing cut-off based indices. One of the limitations, as noted in the literature [2], [3], [8], with the diet quality measures is their lack of ability to predict health outcomes. However, in our recent work we showed that CFUI predicts outcomes [31]. In future work, we will examine weighting of CFUI components. To acknowledge, the CFUI is one of the few indices that enable assessment of complementary feeding quality [7], a nutritionally and behaviourally important period. With its methodological advances and demonstrated associations with health and development outcomes in childhood, the CFUI can be used to guide the development and evaluation of early life nutrition promotion activities.

## Supporting Information

File S1 Contains the following information: Appendix S1: Fourteen components included in the index. Appendix S2: Theorem: Between two paths, the path closer to ideal path will give a higher CFI-DI score. Figure S1.
(DOC)
