# Supplementary material for: A Preference Based Measure of Complementary Feeding Quality: Application to the Avon Longitudinal Study of Parents and Children
Source: PLoS One. 2013 Oct 14;8(10):e76111. doi: 10.1371/journal.pone.0076111 (PMC3796535; doi:10.1371/journal.pone.0076111)
Supplement: File S1 — Contains the following information: Appendix S1: Fourteen components included in the index. Appendix S2: Theorem: Between two paths, the path closer to ideal path will give a higher CFI-DI score. Figure S1. (DOC) [file pone.0076111.s001.doc]

**Appendix S1**

| Fourteen components included in the index |
| --- |
| 1. Breastfeeding duration, 2. Feeding on demand, 3. Timing of solids introduction, 4. Exposure to iron-rich cereals, 5. Frequent exposure to vegetables, 6. Frequent exposure to fruits, 7. Protein foods variety, 8. Exposure to type of sugary drinks, 9. Exposure to confectionary, cakes, biscuits, and savoury snacks, 10. Timing of cow milk introduction, 11. Exposure to tea, 12. Timing of lumpy foods introduction, 13. Exposure to commercial infant foods, 14. Number of daily meals and snacks |

**Appendix S2:**

Theorem: Between two paths, the path closer to ideal path will give a higher CFI-DI score.

Proof: Take a cross-sectional view of the CFI space (given in Figure S1) and prove this using the sin law.

Let *BF_1_*, *V_1_*, *SD1* be the base position and *BF*_2_, *V*_2_, *SD*_2_, be a higher point on the ideal path. The distance between these two points be defined as $d_{12}=\sqrt{\left(BF_{2}-BF_{1}\right)^{2}+\left(V_{2}-V_{1}\right)^{2}+\left(SD_{2}-SD_{1}\right)^{2}}.$ The locus of all points at the same distance is a sphere having radius *d*_12_ and the centre are *BF*_1_, *V*_1_, *SD*_1_. Consider the point on the sphere at *BF*_3_, *V*_3_, *SD*_3_ so that *d*_12_ = *d*_13_ and the joining of these two lines makes an angle, $\theta$. Let *d*_j1_ denote the distance joining the *j*th point with the ideal point note that (*d*_1I_ = *d*_12_+*d*_2I_) ; Applying the law of sine’s to the triangle we now show that the path closer to ideal path will have higher CFI- DI score.

| $\frac{d_{3I}}{\sin\theta}=\frac{d_{12}}{\sin\gamma}=\frac{d_{12+2I}}{\sin\left(\pi-\left(\theta+\gamma\right)\right)}$ | (**A-1**) |
| --- | --- |

as$\sin\left(\pi-\left(\theta+\gamma\right)\right)$=$\sin\left(\theta+\gamma\right)$, substituting this in equation A-1 leads to

$$\frac{d_{3I}}{\sin\theta}=\frac{d_{12}}{\sin\gamma}=\frac{d_{12+2I}}{\sin\left(\theta+\gamma\right)}$$

We now solve for $\gamma$, by manipulating the above equation, this can be re-written as

$$\frac{\sin\left(\theta+\gamma\right)}{\sin\gamma}=\frac{d_{12+2I}}{d_{12}}$$

Now using the formula that $\sin\left(\theta+\gamma\right)=\cos\theta\sin\gamma+\cos\gamma\sin\theta$this equation can be written as

$$\begin{matrix} \tan\gamma=\frac{d_{12}\sin\theta}{d_{12}\left(1-\cos\theta\right)+d_{2I}} \\ \gamma=\tan^{-1}\left(\frac{d_{12}\sin\theta}{d_{12}\left(1-\cos\theta\right)+d_{2I}}\right), \end{matrix}$$

Using the rule $\tan^{-1}(x)=\sin^{-1}\left(\frac{x}{\sqrt{x^{2}+1}}\right)$ the above equation can now be written as a function of sine and this is

$$\gamma=\sin^{-1}\left(\frac{d_{12}\sin\theta}{\sqrt{2d_{12}^{2}\left(1-\cos\theta\right)+d_{2I}^{2}}}\right),$$

Substituting the value of $\gamma$in equation A.1 and solving for d_3I_ gives

| $\begin{matrix} \frac{d_{3I}}{\sin\theta}=\frac{d_{12}}{\sin\gamma} \\ d_{3I}=\frac{d_{12} \sin\theta}{\sin\left(\sin^{-1}\left(\frac{d_{12}\sin\theta}{\sqrt{2d_{12}^{2}(1-\cos\theta)+d_{2I}^{2}}}\right)\right)} \\ d_{3I}=\sqrt{2d_{12}^{2}(1-\cos\theta)+d_{2I}^{2}} \end{matrix}$ | (**A-2**) |
| --- | --- |

Squaring both sides of A-2 and differentiating with respect to *θ* $\frac{\partial\left(d_{3I}^{2}\right)}{\partial\theta}=2d_{12}^{2}\sin\theta$, for $\theta\in\left(0,\pi\right)$, sinθ >0 and hence *d*_3I_ is an increasing function of θ. This proves that between two paths, the path closer to ideal path will give a higher *CFI^DI^*.


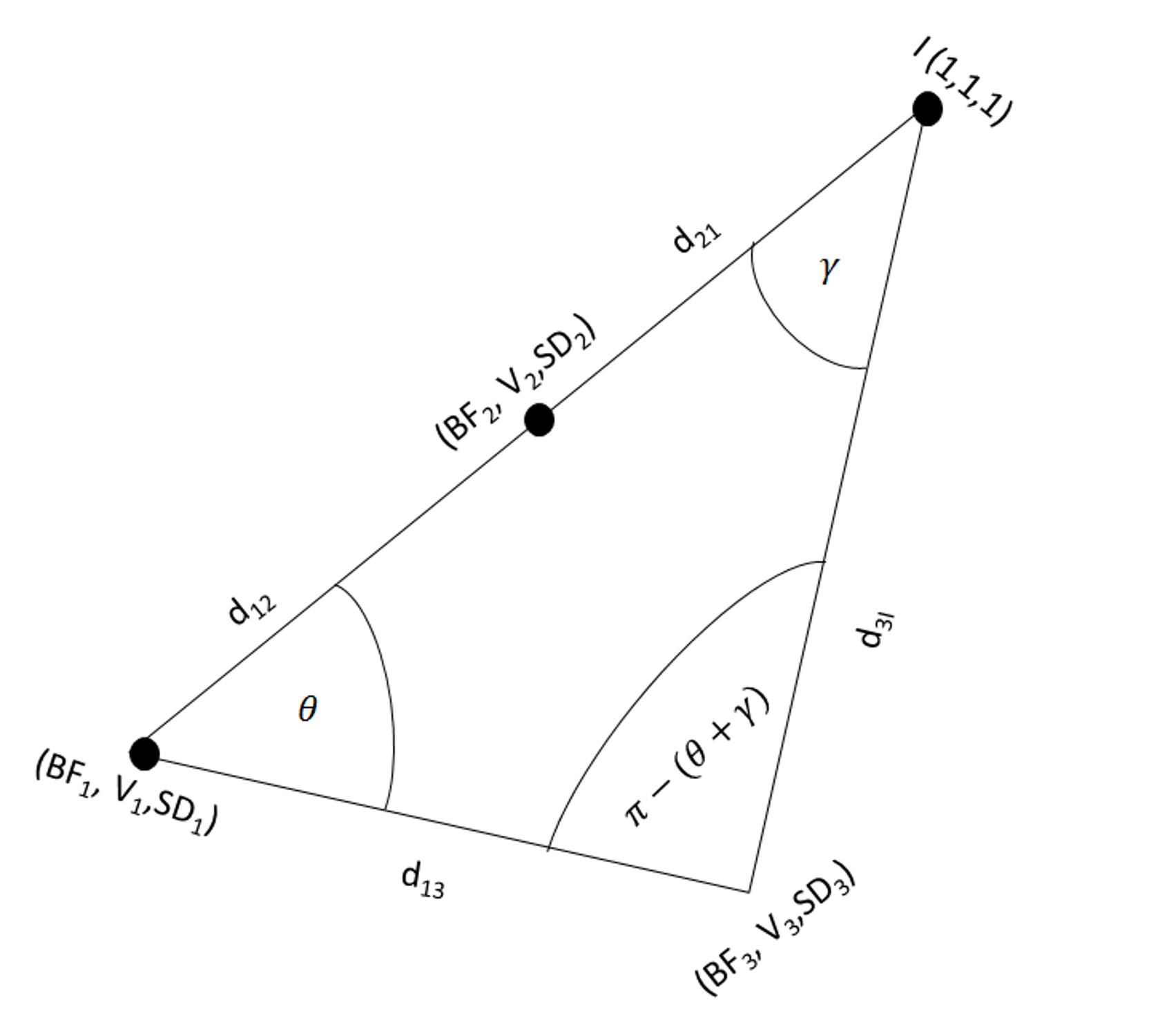


Figure S1 Cross sectional view of the CFUI- DI space

**Footnote: (BF_i_-Breastfeeding V_i_-Vegetable SD_i_- Sugary Drinks, i =1, 2, 3)**
